# Genome-Wide Analysis of the *Rad21*/*REC8* Gene Family in Cotton (*Gossypium* spp.)

**DOI:** 10.3390/genes14050993

**Published:** 2023-04-27

**Authors:** Yali Wang, Lili Zhou, Huiming Guo, Hongmei Cheng

**Affiliations:** 1 Biotechnology Research Institute, Chinese Academy of Agricultural Sciences, Beijing 100081, China; wangyali_bio@163.com (Y.W.); zhoulilinew@163.com (L.Z.); 2 National Nanfan Research Institute, Chinese Academy of Agricultural Sciences, Sanya 572024, China

**Keywords:** cohesin, cotton, gene expression, stress, bio-information analysis

## Abstract

Cohesin is a ring-shaped protein complex and plays a critical role in sister chromosome cohesion, which is a key event during mitosis and meiosis. Meiotic recombination protein REC8 is one of the subunits of the cohesion complex. Although *REC8* genes have been characterized in some plant species, little is known about them in *Gossypium*. In this study, 89 *REC8* genes were identified and analyzed in 16 plant species (including 4 *Gossypium* species); 12 *REC8* genes were identified in *Gossypium. hirsutum*, 11 in *Gossypium. barbadense*, 7 in *Gossypium. raimondii,* and 5 in *Gossypium. arboreum*. In a phylogenetic analysis, the 89 *RCE8* genes clustered into 6 subfamilies (I–VI). The chromosome location, exon-intron structure, and motifs of the *REC8* genes in the *Gossypium* species were also analyzed. Expression patterns of *GhREC8* genes in various tissues and under abiotic stress treatments were analyzed based on public RNA-seq data, which indicated that *GhREC8* genes might have different functions in growth and development. Additionally, qRT-PCR analysis showed that MeJA, GA, SA, and ABA treatments could induce the expression of *GhREC8* genes. In general, the genes of the *REC8* gene family of cotton were systematically analyzed, and their potential function in cotton mitosis, meiosis, and in response to abiotic stress and hormones were preliminary predicted, which provided an important basis for further research on cotton development and resistance to abiotic stress.

## 1. Introduction

Cohesion, a muti-subunit protein complex, is essential for sister chromatid cohesion and chromatin loop structure in mitosis and meiosis [1]. The loss and mutation of cohesion can cause abnormal chromosomal separation or alloploidy gamete production. Genes involved in the regulation of meiosis have been extensively studied in Arabidopsis, rice, maize and many other plants [2,3,4]. Meiotic recombination protein REC8, one of the subunits of the meiosis-specific cohesion protein complex, is conserved in functional evolution from yeast to humans. Previous studies have shown that the *REC8* gene is required for correct meiosis. During meiosis in *Saccharomyces cerevisiae* and *Schizosaccharomyces pombe*, REC8 was found in the cohesion complex, which has homology to SCC 1/Mcd 1/Rad 21 proteins [5,6,7]. *REC8* mutations in *S. cerevisiae* and *S. pombe* disrupt the cohesion of sister chromosome arms and centromeres, resulting in reduced recombination, affecting the formation of the synaptonemal complex and causing premature separation of sister chromatids during the first meiotic division [5,7,8,9]. Subsequently, proteins homologous to yeast REC8 have been found in other species, including mice, humans [6,10], *Caenorhabditis elegans* [11], *Arabidopsis thaliana* [12,13], *Zea mays* [14], *Drosophila* [15], *Xenopus laevis* [16], *Oryza sativa* [17], and *Citrullus lanatus* [18]. In *C. elegans*, RNAi of *REC8* leads to univalent formation and chromosomal fragmentation at diakinesis [11]. In *Arabidopsis*, *syn1*/*dif1* plants exhibit defects in chromosome cohesion and condensation during meiosis, resulting in chromosome fragmentation and the formation of microsporophyll [19,20]. In maize, absence of first division (AFD1) is a homologous protein of REC8, and mutations in *AFD1* affect leptotene chromosomes [14]. Different homologs of REC8 are present in different species, and a species may even have several homologs. The first protein identified in this protein family was fission yeast Rad21, which is involved in the repair of DNA double-bond breaks [21]. REC8 is not only required for cohesion between sister chromatids [22] but also for the assembly of synaptonemal complexes [23], organization of meiotic chromatid structure [24], and homologous chromosome recombination [25]. It is also associated with the synapsis of homologous chromosomes and plays the same role in plant cells [26].

Cotton is not only a fiber and oil crop but also a high-protein food crop and important strategic material. Homologous recombination that takes place during meiosis (using the principle of homologous recombination) is a critical process for breeding and developing new varieties with traits for high yield, disease resistance, and ornamental appeal, so the study of genes related to the meiosis process is of great biological and economic significance [27]. *REC8* genes have been identified in many species; however, few studies on their functions in cotton have been reported, especially in the mechanism of fertility and the development process. In the present study, 89 *REC8* genes in 16 species were identified and analyzed. Among them, 35 cotton *REC8* genes were characterized, and structural diversity, phylogenetic relationships, the chromosomal distribution, and expression levels in different tissues and under different abiotic stresses were investigated. Our research provides insights that will help develop a comprehensive understanding of the biological functions of the cotton *REC8* gene family in fertility and responses to various stresses.

## 2. Materials and Methods

### 2.1. Identification of REC8 Gene Family Members in Gossypium Species

Genome sequences file of *G. arboretum* (CRI, version 1.0), *G. raimondi* (JGI, version 2.0), *G. hirsutism* (ZJU, version 1.0), and *G. barbadense* (ZJU, version 1.0) were downloaded from COTTONGENE (http://www.cottongen.org/ (accessed on 30 June 2022)) and the Cotton Functional Genomics Database (https://cottonfgd.org/ (accessed on 29 June 2022)). The *Arabidopsis REC8* genome file was obtained from The *Arabidopsis* Information Resource (TAIR, https://www.Arabidopsis.org/ (accessed on 22 June 2022)). Genomic data for *Zea mays*, *Oryza sativa*, *Sorghum bicolor*, *Ananas comosus*, *Vitis vinifera*, *Populus trichocarpa*, *Theobroma cacao*, *Glycine max,* and *Amborella trichopoda* were downloaded from the Ensemble database (http://plants.ensembl.org/index.html (accessed on 6 August 2022)). Genome sequence data of *Volvox carteri* and *Chlamydomonas reinhardtii* were downloaded from the National Center for Biotechnology Information (NCBI).

We used AtREC8 amino acid sequences as query sequences to search the four cotton species’ protein databases for candidate sequences by the blastp program (E-value < 1 × 10^−5^). Candidate amino acid sequences were determined using the Hmmsearch program and Conserved Domain Database (CDD) in NCBI to search for the REC8 domain (PF04824 and PF04825) with default parameters. REC8s in *Zea mays*, *Oryza sativa*, *Sorghum bicolor*, *Ananas comosus*, *Vitis vinifera*, *Populus trichocarpa*, *Theobroma cacao*, *Glycine max,* and *Amborella trichopoda* were identified following the same process.

Then, the physical and chemical properties of *REC8* genes of cotton were analyzed using the online tool ExPASy (http://web.expasy.org/ (accessed on 22 September 2022)), and the subcellular localization of these genes were predicted by Cell-PLoc (http://www.csbio.sjtu.edu.cn/bioinf/Cell-PLoc-2/ (accessed on 4 January 2023)).

### 2.2. Phylogenetic Analysis

The REC8 protein sequences from 16 species were aligned using MUSCLE, and the neighbor-joining (NJ) phylogenetic tree of 16 species was constructed using MEGA 7 [28], with a bootstraps value of 1000 and other parameters set to the default value. The resulting tree was then decorated using iTOL (https://itol.embl.de/upload.cgi (accessed on 26 October 2022)).

### 2.3. Chromosomal Locations and Collinearity Analysis of Cotton REC8 Genes

The chromosome location information of four cotton species (*G. arboretum, G. raimondii, G. hirsutum,* and *G. barbadense*) was obtained from the reference cotton database. The distribution of *REC8* genes on the four cotton chromosomes was drawn using TBtools [29]. The multiple collinearity scan (MCS-scanX) in TBtools was used to analyze the collinearity of *REC8* genes. The collinearity map was drawn by using Basic CIRCOS in TBtools (v1.108).

### 2.4. Calculation of Selection Pressure for Duplicated Gene Pairs

CDS sequences of cotton *REC8* genes were obtained from the reference cotton database, and then the non-synonymous (Ka) and synonymous substitution rates (Ks) were calculated using TBtools software (v1.108). A Ka/Ka ratio > 1 means positive selection, a Ka/Ka ratio = 1 indicates neutral selection, and a Ka/Ka ratio < 1 represents negative selection.

### 2.5. Gene Structure, Motif, and Domain Analysis

The conserved motifs of REC8 proteins were identified using Multiple Em for Motif Elicitation (MEME) (http://memesuite.org/ (accessed on 25 June 2022)) with the *p*-value of each motif on each protein being lower than 1 × 10^−5^. The exon–intron structure of *REC8* genes was analyzed using TBtools. Phylogenetic trees with motif, domain, and gene structure were visualized using TBtools.

### 2.6. Analysis of Cis-Acting Elements in REC8 Promoters

The 2000 bp upstream promoter sequences of *REC8* genes were extracted. *cis*-acting elements were then predicted by PlantCare (http://bioinformatics.psb.ugent.be/webtools/plantcare/html/ (accessed on 28 September 2022)). A phylogenetic tree with identified elements was visualized using TBtools.

### 2.7. Expression Profile of REC8 Genes under Different Tissues and Stress

To analyze the expression pattern of *REC8* genes under different tissues and stress (heat, cold, salt, and PEG), the transcriptome data were downloaded from the Cotton Omics Database (http://cotton.zju.edu.cn/10.rnasearch (accessed on 2 July 2022)) [30]. The heat map was drawn using the TBtools software (v1.108) by using fragments per kilo base of exon per million mapped (FPKM).

### 2.8. Plant Materials and Treatments

*G. hirsutum* cv. Coker 312 (R15) was grown in a chamber (28 °C, 16/8 h day/night). Plants at the third-leaf stage were treated with 100 μM MeJA, 2 mM SA, and 200 μM ABA. The leaves of treated plants were collected at 0 h, 3 h, 6 h, and 12 h and were frozen in liquid nitrogen immediately for further analysis.

### 2.9. RNA Extraction and qRT-PRC Analysis

Total RNA was extracted from the frozen leaves using RNAprep Pure Plant Kit (TianGen, Beijing, China). HiScript III Reverse Transcriptanse (Vazyme, Nanjing, China) was used to convert RNA to cDNA. The qPCR analysis was performed using Taq Pro Universal SYBR qPCR Master Mix (Vazyme, Nanjing, China) on the ABI 7500 Fast Real-Time PCR system (Thermo, Waltham, MA, USA). Three biological replicates were performed in all analyses. *GhUBQ7* was used as the internal reference gene, and the data were analyzed using the 2^−∆∆Ct^ method [31]. The primers are listed in Table S6.

## 3. Results

### 3.1. Identification of REC8 Genes in Cotton

To identify the *REC8* genes in the 4 cotton species and the other 12 species, we selected candidate genes from a search and comparison using HMMsearch and blastp programs. Then, the obtained sequences were filtered via CDD. Listed in Table S1 are the 12 *REC8* genes found in *G. hirsutum*, 11 in *G. barbadense*, 7 in *G. raimondii*, 5 in *G. arboretum*, 3 in *A. comosus*, 4 in *Arabidopsis*, 7 in maize, 4 in rice, 4 in *A. trichopoda*, 9 in *P. trichocarpa*, 6 in *G. max*, 5 in *S. bicolor*, 7 in *T. cacao*, 3 in *V. vinifera*, 1 in *V. carteri,* and 1 in *C. reinhardtii*. Twice as many *REC8* genes were found in *G. hirsutum* and *G. barbadense* as in *G. raimondii* and *G. arboretum*, which indicated that the tetraploid cotton is obtained by hybridizing the diploid A genome and the diploid D genome, followed by chromosome doubling. In addition, the characteristics of REC8 proteins in cotton were analyzed, including the protein sequence size, molecular weight (MV), isoelectric point (pI), and instability coefficient (Table S2). The length of 35 REC8 proteins ranged from 529 aa to 1193 aa; GbREC8-02-D had the fewest amino acids, and GhREC8-03-D and GrREC8-02 had the most. The molecular weight was between 59,052.57 and 130,250.69 kD and was lowest for GbREC8-02-D and highest for GhREC8-03-D. The pI ranged from 4.29 to 7.32; two proteins had a pI less than 7, and 33 had a pI greater than 7, indicating that most of the REC8 proteins were alkaline. The REC8 proteins were predicted to be localized in the nucleus where the REC8 proteins worked probably (Table S2).

### 3.2. Phylogenetic Analysis of the Cotton REC8 Gene Family

**Figure 1:**
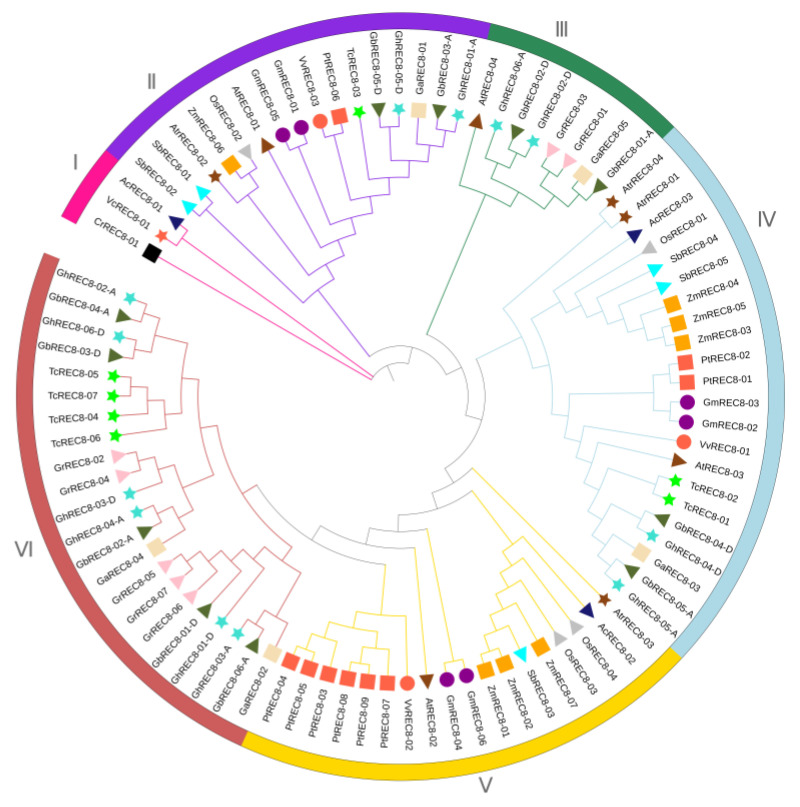
Phylogenetic relationships of 89 identified *REC8* genes from 4 cotton species and 12 other plant species. Six subfamilies (I–VI) are colored in different colors. Different color shapes represent different species. The neighbor-joining (NJ) phylogeny tree was created using MEGA 7 (bootstrap value = 1000).

To further study the evolutionary relationship of the *REC8* gene family in plants, 89 REC8 protein sequences from 4 cotton species and 12 other species were used to construct the phylogenetic NJ tree using MEGA 7 software. In the phylogenetic tree, the REC8 proteins were clustered into 6 subfamilies (I–VI) (Figure 1) with 3 members in subfamily I, 16 in II, 8 in III, 22 in IV, 18 in V, and 22 in VI. Except for subfamilies III and VI, the other four subfamilies were composed of *REC8* genes from monocotyledon and dicotyledons, indicating that REC8 proteins were highly conserved. Subfamily III comprised *REC8* genes from Arabidopsis and the four cotton species, and subfamily VI comprised *REC8* genes from *T. cacao* and the four cotton species. We found that the *REC8* genes of cotton were more closely related to those of *T. cacao*, since the *REC8* genes of cotton and cacao were closely clustered in the phylogenetic tree. In subfamilies II and IV, one *TcREC8* gene corresponded to five homologous *REC8* genes from three *Gossypium* species: one from *G. arboretum*, two from *G. hirsutum*, and two from G. barbadense. In addition, *AtREC8-04* has a role in DNA repair [32], and loss of function in *AtREC8-01* and *AtREC8-03* affects meiosis [12,20,33]. *GhREC8-06-A*, *GhREC8-02-D*, *GbREC8-02-D*, *GbREC8-01-A*, *GrREC8-03*, *GrREC8-01,* and *AtREC8-04* in subfamily III were on the same branch, indicating that they may be involved in DNA repair. *GhREC8-04-D*, *GhREC8-05-A*, *GbREC8-04-D*, *GbREC8-05-A*, and *GaREC8-03* in subfamily IV may have similar functions as *AtREC8-03*.

### 3.3. Chromosomal Distribution and Synthesis Analysis of REC8 Genes in Cotton

**Figure 2:**
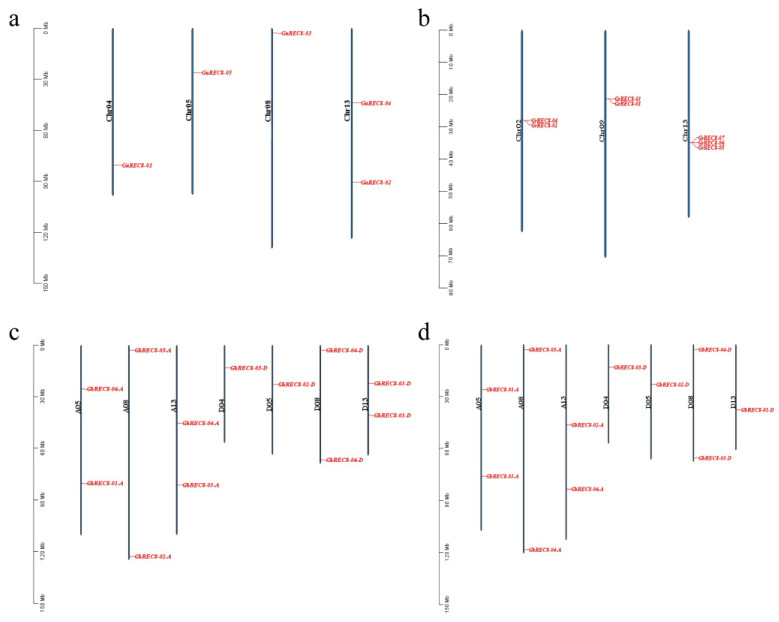
Distribution of *REC8* genes on chromosomes of (**a**) *G. arboretum*, (**b**) *G. raimondii*, (**c**) *G. hirsutum,* and (**d**) *G. barbadense*. Chromosome numbers are shown on the left of the chromosomes. *REC8* genes are labeled on the right of the chromosomes. Scale bar on the left indicates the chromosome lengths (Mb).

Based on the genomes of the four cotton species, we analyzed the location of the *REC8* genes on the chromosomes. One *GaREC8* gene was on each of three chromosomes (Chr04, Chr05, and Chr08), and two were on Chr13 (Figure 2a). Two *GrREC8* genes were mapped on each of two chromosomes (Chr02 and Chr09), and Chr13 had three (Figure 2b). The chromosome distribution of *REC8* genes was highly similar between *G. hirsutum* and *G. barbadense* (Figure 2c,d). The *G. hirsutum* genome had 12 *GhREC8* genes among 7 chromosomes (1 each on chromosomes D04 and D05; 2 each on A05, A08, A13, D08, and D13). The *G. barbadense* genome had 11 *GbREC8* genes among 7 chromosomes (1 each on D04, D05, and D13; 2 each on A05, A08, A13, and D08).

**Figure 3:**
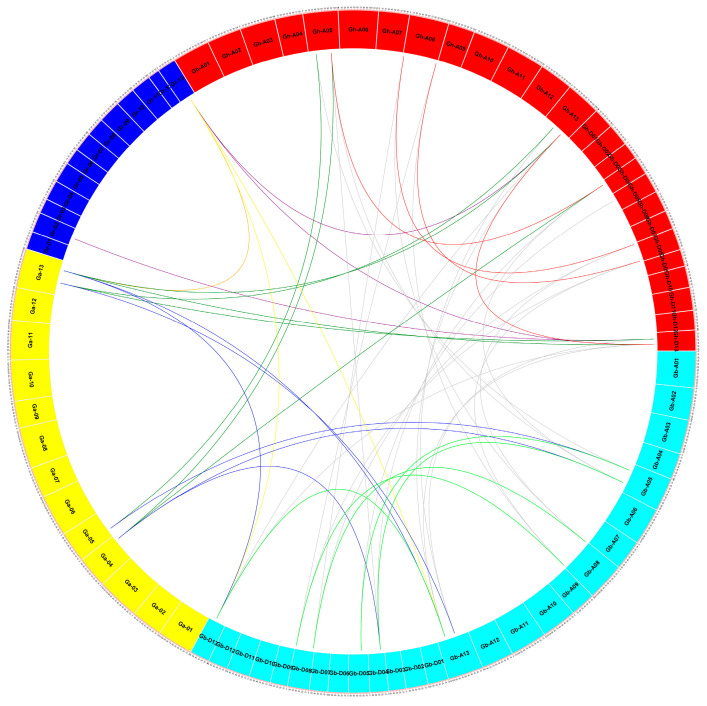
Syntenic analysis of *REC8* genes from four cotton species. Chromosomes of *G. arboretum* are in yellow, *G. raimondii* in dark blue, *G. hirsutum* in red, and *G. barbadense* in light blue.

To elucidate the relationship of *REC8* orthologs in cotton, we performed a synthetic analysis on four cotton species. A total of 35 *REC8* genes of cotton have synthetic relationships, including 12 from *G. hirsutum*, 11 from *G. barbadense*, 7 from *G. raimondii,* and 5 from *G. arboretum* (Figure 3, Table S3). There were 47 orthologous/paralogous gene pairs: 4 gene pairs between *G. hirsutum* and *G. hirsutum*, 20 pairs between *G. hirsutum* and *G. barbadense*, 3 between *G. hirsutum* and *G. raimondii*, 7 between *G. hirsutum* and *G. arboretum*, 4 between *G. barbadense* and *G. barbadense*, 6 between *G. barbadense* and *G. arboretum*, 2 between *G. barbadense* and *G. raimondii,* and 1 between *G. arboretum* and *G. raimondii*. These results indicated that *REC8* genes in *G. hirsutum* and *G. barbadense* had a close evolutionary relationship. Then, we analyzed the collinear relationship of *REC8* genes between *G. hirsutum* and two species (*A. thaliana* and *T. cacao*). The results showed that the number of orthologous gene pairs between *G. hirsutum* and *A. thaliana* and *T. cacao* was 2 and 5, respectively (Figure S1, Table S4). Very few collinear gene pairs of *G. hirsutum* and the two species were anchored to the highly conserved synthetic blocks.

We calculated the Ka/Ks ratio of 47 duplicated *REC8* gene pairs from 8 combinations of 4 cotton species (*G. hirsutum* vs. *G. hirsutum*, *G. barbadense* vs. *G. barbadense*, *G. barbadense* vs. *G. arboretum*, *G. barbadense* vs. *G. raimondii*, *G. hirsutum* vs. *G. arboretum*, *G. hirsutum* vs. *G. barbadense*, *G. hirsutum* vs. *G. raimondii,* and *G. arboretum* vs. *G. raimondii*) (Table S5). The results showed that the Ka/Ks ratios from 2 gene pairs (*GhREC8-04-D/GbREC8-04-D* and *GhREC8-06-D/GbREC8-03-D*) were larger than 1, indicating that these gene pairs experienced rapid evolution. A total of 23 gene pairs had a Ka/Ks ratio less than 0.5, and 22 gene pairs were between 0.5 and 1, leading us to hypothesize that these gene pairs have undergone strong purifying selection.

### 3.4. Structure Analysis of Cotton REC8 Genes

**Figure 4:**
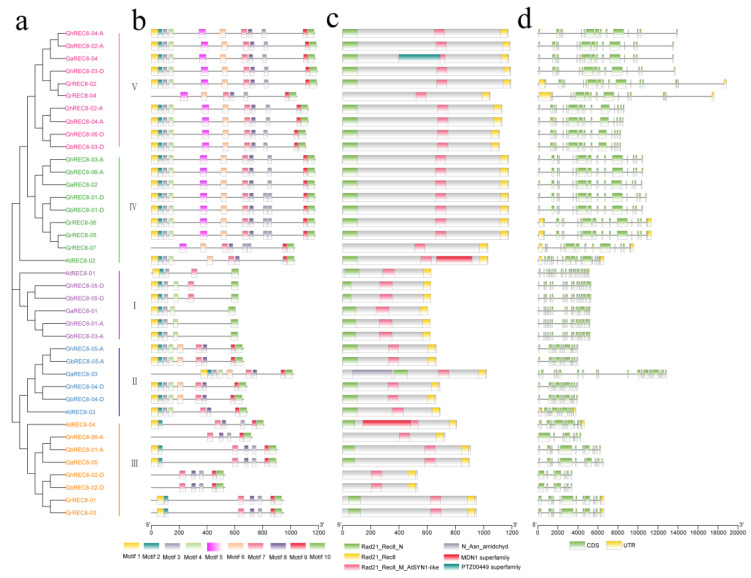
Phylogenetic relationship, motif, domain, and gene structure of *REC8* genes from cotton and *Arabidopsis*. (**a**) The NJ phylogenetic tree was constructed using MEGA 7 software based on the REC8 protein sequences from four cotton species and *Arabidopsis*. Five clusters (I–V) are labeled in different colors. (**b**) Motif composition of REC8 proteins from four cotton species and *Arabidopsis*. The 10 motifs are displayed in different colored boxes. The scale bar at the bottom represents the length of REC8 amino acid sequences (aa). (**c**) Conserved domain in REC8 proteins from four cotton species and *Arabidopsis*. Different domains are labeled in different colors. The scale bar at the bottom represents the length of REC8 amino acid sequences (aa). (**d**) Intron/exon structure of *REC8* genes from four cotton species and *Arabidopsis*. CDS and UTR are displayed in green and yellow boxes, respectively. The scale bar at the bottom represents the length of *REC8* genomic sequences (bp).

Structural differences in genes play an important role in the gene evolution of a gene family. To better understand the structure of *REC8* genes in cotton, we analyzed the sequence structure, motif, and conserved domains of cotton *REC8* genes based on the phylogenetic tree (Figure 4a). Ten motifs (named motifs 1 to 10) were predicted in the REC8 proteins by using MEME. Common motifs and similar motif arrangements were found in most REC8 proteins within the same subfamily, suggesting that the structure was conserved in these proteins. Eleven motifs were found in the proteins of subfamily V and IV except for GrREC8-04 and GrREC8-07. Motif 2 and motif 4 were absent in GhREC8-06-A, GhREC8-02-D, and GbREC8-02-D in subgroup III (Figure 4b). Whether these specific motifs have an effect on the function of REC8 proteins remains to be studied. Most cotton REC8 proteins had a similar domain to *Arabidopsis* REC8 except that GrREC8-04, GrREC8-07, GhREC8-06-A, GhREC8-02-D, and GbREC8-06-A lacked the N-segment Rad21_REC8 domain. AtREC8-02 in subgroup IV and AtREC8-04 in subgroup III had the MDN1 domain, which was not observed in REC8 proteins from cotton in the same subgroup. In addition, PTZ00449 and N_Asn amidohyd domains were found in GaREC8-04 and GaREC8-03, respectively, indicating that these domains might confer protein different biological functions (Figure 4c). Motifs 1, 2, 3, and 4 were in the N-terminal domain; motifs 9 and 10 were in the C-terminal domain.

Then, we analyzed the intron/exon structure of the *REC8* genes. The structures were similar for *REC8* genes in the same subclade, especially among genes in subfamily IV with 15 exons, indicating that *REC8* gene structures are conserved among 4 species. However, 1 gene (*GaREC8-03*) in subclade II had 22 exons, 1 (*GhREC8-04-D*) had 11 exons, and the other genes had 12 exons. In subclade III, 4 *REC8* genes in cotton (*GbREC8-01-A*, *GaREC8-05*, *GrREC8-01,* and *GrREC8-03*) and 1 in *A. thaliana* (*AtREC8-04*) had 13 exons, and 3 in cotton (*GhREC8-06-A*, *GhREC8-02-D,* and *GbREC8-02-D*) had 8 exons (Figure 4d). These results suggested that genes with different exon/intron numbers might have special biological functions.

### 3.5. Analysis of Cis-Acting Elements of the REC8 Genes in Cotton Species

**Figure 5:**
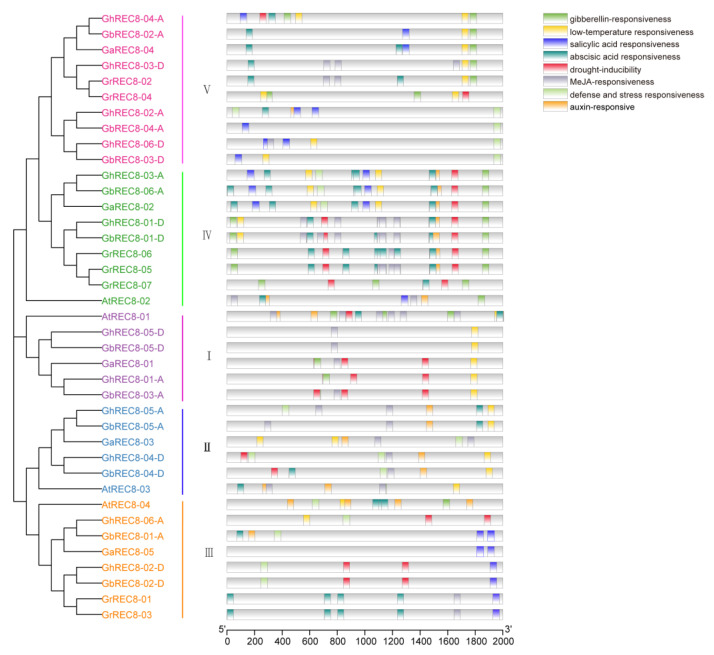
*Cis*-acting element analysis of promoters of *REC8* genes from four cotton species and *Arabidopsis*. Different colored boxes represent different *cis*-acting elements. An NJ phylogenetic tree on the left from REC8 protein sequences was constructed using MEGA7.0. The scale bar at the bottom represents the length of promoters (bp).

Gene expression is regulated by upstream promoters, where many *cis*-acting elements are found. To better understand the regulation and potential function of the target genes, we extracted the 2000 bp upstream sequence from the predicted translation start site of each *REC8* gene to analyze the *cis*-acting elements. *Cis*-acting elements involved in hormone (IAA/auxin, GA (gibberelins), MeJA (methyl jasmonate), SA (salicylic acid), and ABA (abscisic acid)) and stress responses (low temperature, drought, and defense) and development were predicted using the PlantCARE tool. Promoters of 14 genes contained an IAA-responsive element, 17 contained a GA-responsive element, 16 contained an SA-responsive element, 20 contained an ABA-responsive element, and 19 contained a MeJA-responsive element. Among stress elements found in the *REC8* promoters, low-temperature response elements were found in promoters of 24 *REC8* genes, drought response elements in those of 18, and defense and stress response elements in those of 15. Seven genes (*GbREC8-03-A*, *GbREC8-01-D*, *GhREC8-01-A*, *GhREC8-04-A*, *GhREC8-01-D*, *GrREC8-04,* and *GaREC8-01*) involved in low-temperature and drought responsiveness were found (Figure 5). These results showed that *REC8* genes played a role in hormone, stress, and defense responses in cotton.

### 3.6. GhREC8 Gene Profiles in Different Tissues of Upland Cotton

**Figure 6:**
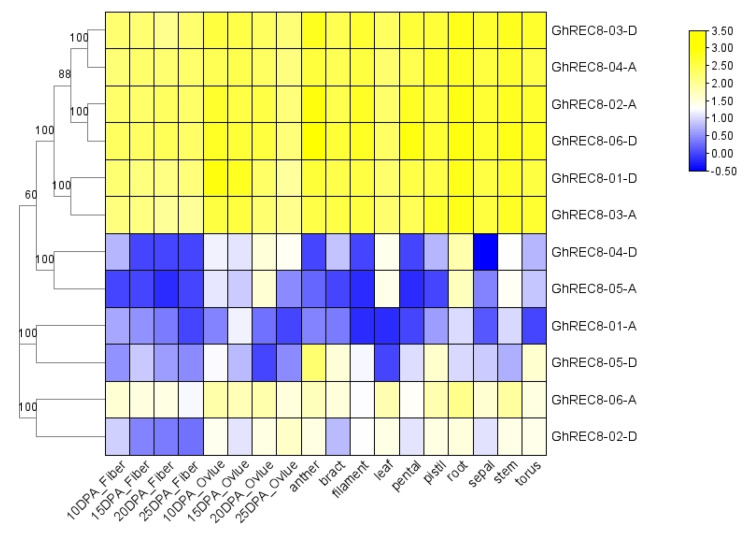
Expression pattern of *GhREC8* genes in different issues. The heat map was drawn based on RNA-Seq data using TBtools. Yellow and blue represent high and low expression levels, respectively. Scale bar represents log_2_^(FPKM)^.

To evaluate the potential role of the 12 *GhREC8* genes in growth and development, publicly available transcriptome data were used to analyze gene expression patterns in fibers, ovules, anthers, bracts, filaments, leaves, petals, pistils, roots, sepals, stems, and torus (Figure 6). Six *GhREC8* genes (*GhREC8-03-D*, *GhREC8-04-A*, *GhREC8-02-A*, *GhREC8-06-D*, *GhREC8-01-D*, *GhREC8-03-A,* and *GhREC8-06-A*) were highly expressed in almost all tissues and stages. *GhREC8-04-D* and *GhREC8-05-A* were similar in being highly expressed during ovule development in leaves, roots, and stems. There were high levels of *GhREC8-05-D* expression in anthers, bracts, the filament, pistils, and torus. Compared with fiber development, *GhREC8-02-D* showed relatively higher expression in other issues and stages. These results indicated that most *GhREC8* genes were expressed in vegetative tissues.

### 3.7. Expression Patterns of GhREC8 Genes under Abiotic Stress and Hormone Treatments

**Figure 7:**
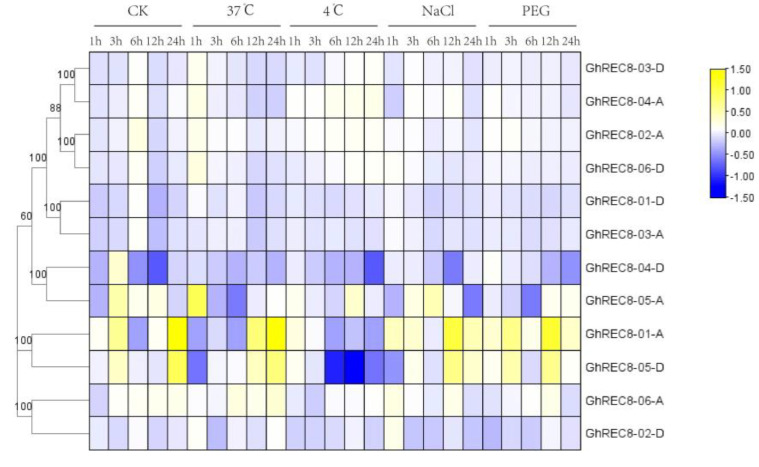
Expression level of *GhREC8* genes under different stresses (37 °C, 4 °C, NaCl, and PEG). The heat map was drawn based on RNA-Seq data using TBtools. Yellow and blue represent high and low expression levels, respectively. Scale bar represents log_2_^(FPKM)^.

**Figure 8:**
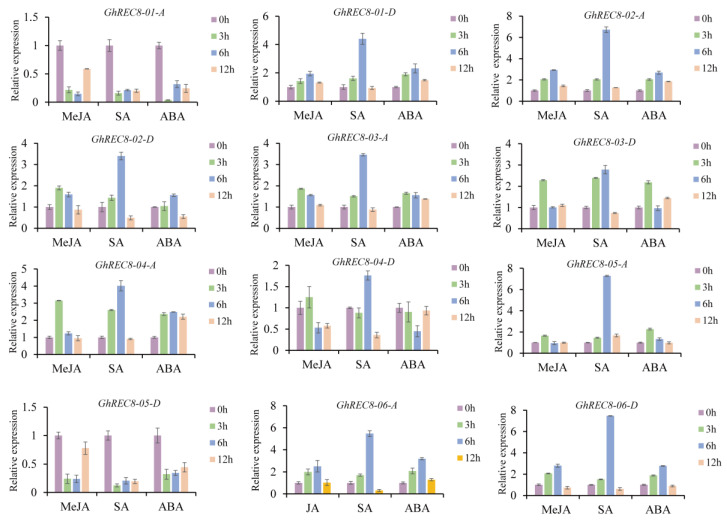
Expression level of *GhREC8* genes in leaves after MeJA, SA, and ABA treatments for 0, 3, 6, and 12 h. Data represent means (±SD) of three biological replicates.

To explore the potential function of the *REC8* genes in response to abiotic stresses in upland cotton, we used public transcriptome data to analyze the effect of stresses from high temperatures, low temperatures, PEG, and salt and treatment with hormones (Figure 7). The expression of most *GhREC8* genes did not change significantly after abiotic stress treatments. *GhREC8-01-A* and *GhREC8-05-D* were upregulated after high-temperature, NaCl, and PEG treatments, especially at 12 h and 24 h after treatments. However, *GhREC8-01-A and GhREC8-05-D* were downregulated after exposure at 4 °C. *GhREC8-05-A* was induced by high temperatures, 4 °C, NaCl, and PEG with respective peaks in expression at 1 h, 12 h, 6 h, and 24 h. After MeJA, SA, and ABA treatments, most *GhREC8* genes were upregulated, and the expression of three genes (*GhREC8-01-D*, *GhREC8-02-A,* and *GhREC8-06-D*) peaked by 6 h after treatments, and two (*GhREC8-01-A* and *GhREC8-05-D*) were downregulated. *GhREC8-04-D* was upregulated after MeJA and SA treatments, with respective maximum expression at 3 h and 6 h (Figure 8). These results indicated that *GhREC8* might have potential functions in response to phytohormone and abiotic stresses.

## 4. Discussion

Cohesin is a ring-shaped protein complex that is required for sister chromatid cohesion and DBS repair [34,35]. Centromeric sister chromatid cohesion is essential during meiosis. Previous studies have shown that REC8 is crucial for meiosis. The four members (*SYN1–SYN4*) belonging to the *Rad21* gene family in *A. thaliana* have been identified. *SYN2*/*4* are involved in mitosis, while *SYN1* is essential for meiosis [13], and *SYN3* is essential for megagametogenesis [36]. Mutations of *SYN1* (*REC8*) lead to male and female sterility in *A. thaliana* [37]. In rice, an *Osrec8* mutant had abnormal homologous chromatid pairing and telomere bouquets [38]. The germination of pollen grains declined, and fruits were seedless after the knockout of *ClREC8* in watermelon [18]. It can be seen that REC8 protein plays an important role in the fertility of monocots and dicots. Here, we identified 35 *REC8* genes in 4 cotton species, and tetraploid cotton had twice more *REC8* genes than diploid cotton, which reflected the fact that tetraploid cotton descended from the hybridization of A- and D-genome ancestors. Phylogenetic tree analysis showed that *GhREC8* genes (*GhREC8-05-D, GhREC-01-A, GhREC8-06-A, GhREC8-02-D, GhREC8-04-D, and GhREC8-05-A*) were in the same clade as *SYN1-4* (Figure 1, Table S1), indicating that these genes may have a similar function in meiosis and mitosis. Other studies have shown that *REC8* genes were highly expressed in reproductive tissues [1,13,18]. Similarly, most *GhREC8* genes were highly expressed in floral organs (Figure 6). However, whether it could affect meiosis and miosis needs further research.

Heterosis is widely used in agriculture and has made a great contribution to world food security. The artificial creation of apomiesis may be another effective way to achieve heterosis fixation. The production of cloned gametes is one of the key steps in artificial apomiesis. In previous studies, the *REC8* gene was used to produce cloned gametes in rice and *Arabidopsis* [39,40]. *REC8* genes in the same clade as *AtREC8-01* (*AtREC8*) and *OsREC8-02*(*OsREC8*) may be used for cloned gamete production in cotton.

Rad21/Rec8 proteins from different species share a conserved N-terminal domain and C-terminal domain, which are involved in the interaction with Smc1-Smc3 heterodimer during cohesion establishment [40,41]. Similar N- and C-domains were observed in REC8 proteins from *Arabidopsis* and four cotton species (Figure 4c). Moreover, different exon/intron structures were observed in cotton *REC8* genes (Figure 4d), suggesting differences in gene function and RNA splicing of *REC8* gene family members. The promoters of *REC8* genes contained *cis*-acting elements associated with phytohormone, stress, and defense responses (Figure 5). Then, we analyzed the expression of *GhREC8* genes under MeJA, SA, and ABA treatments, and most were upregulated after hormone treatments, especially after SA treatment, indicating these genes might be involved in hormone regulation. ABA could interact with the JA and SA signal pathways [42]. JA acts as a defense hormone and is also involved in male sterility, root growth, leaf senescence, and other processes [43,44]. Because most *GhREC8* genes were induced by MeJA, they may be related to fertility (Figure 8). The abiotic stresses tested also had different effects on *GhREC8* expression. Most genes were not affected by the abiotic stresses, indicating that these genes did not have an important role in plant responses to these stresses. The expression of *GhREC8-01-A*, *GhREC8-05-A,* and *GhREC8-05-D*, however, was sharply induced by PEG (Figure 7), and they had drought response elements in their promoters, suggesting that these genes might be involved in drought tolerance. These mechanisms still need further research. In conclusion, our results indicated that cotton *REC8* genes likely had multiple functions in the hormone and stress responses and development of plants. Our next step is to study the role of the *REC8* genes in cotton fertility and responses to stresses, which may contribute to the achievement of apomiesis and a further understanding of the mechanisms of resistance to stresses.

## 5. Conclusions

In this study, we identified 89 *REC8* family genes in 16 species, and these genes were divided into 6 subfamilies. Tissue-specific expression pattern analysis indicated that *GhREC8* genes were expressed variously in cotton, indicating that they played different roles in cotton growth and development. An analysis of promoter *cis*-elements and expression levels under abiotic stresses and hormone treatments showed that *GhREC8* genes responded to different stimuli. This study provides valuable information about the cotton *REC8* gene family, which lays a foundation for further revealing the biological roles of *REC8* genes in cotton meiosis, mitosis, and stress adaptation.

## Supplementary Materials

The following supporting information can be downloaded at: https://www.mdpi.com/article/10.3390/genes14050993/s1, Figure S1: Synteny analysis of *REC8* genes between *G. hirsutum* and two species (*Arabidopsis thaliana* and *Theobroma cacao*). Table S1: *REC8* genes in different species. Table S2: Characteristics of cotton *REC8* genes. Table S3: Gene pairs in collinearity. Table S4: Gene pairs between *G. hirsutum* and two species. Table S5: Ka versus Ks of gene pairs. Table S6: Primers for qRT-PCR.
