# Supplementary figures and images for: Genome-Wide Analysis of the Rad21/REC8 Gene Family in Cotton (Gossypium spp.)
Source: Genes (Basel). 2023 Apr 27;14(5):993. doi: 10.3390/genes14050993 (PMC10218516; doi:10.3390/genes14050993)

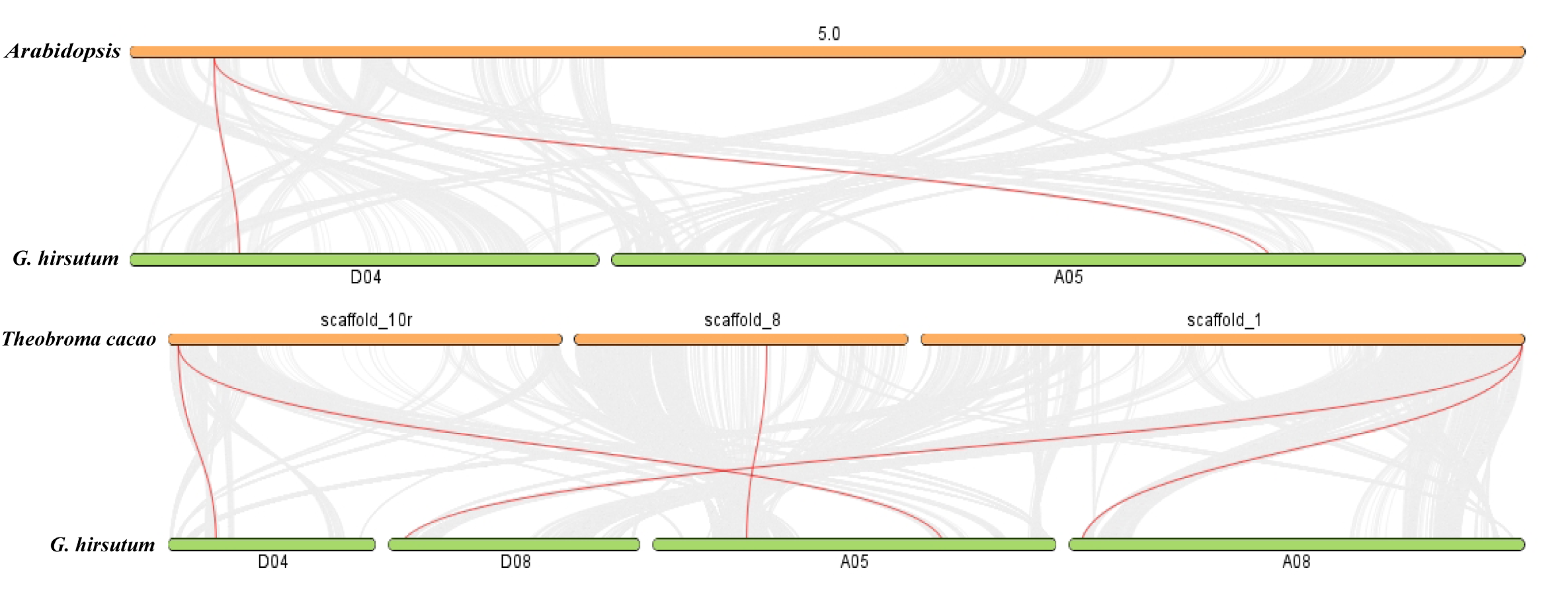

Supplement: Supplementary file 1 [file genes-14-00993-s001.zip › Figure S1.tif]
